# A Critical Review of Recent Advances in Maize Stress Molecular Biology

**DOI:** 10.3390/ijms252212383

**Published:** 2024-11-18

**Authors:** Lingbo Meng, Jian Zhang, Nicholas Clarke

**Affiliations:** 1 School of Geography and Tourism, Harbin University, Harbin 150000, China; zhangjiannefu92@126.com; 2 Norwegian Institute of Bioeconomy Research, 1431 Aas, Norway; nicholas.clarke@nibio.no

**Keywords:** maize, crop stress resistance, molecular biology, stress-resistance genes, yield

## Abstract

With the intensification of global climate change and environmental stress, research on abiotic and biotic stress resistance in maize is particularly important. High temperatures and drought, low temperatures, heavy metals, salinization, and diseases are widespread stress factors that can reduce maize yields and are a focus of maize-breeding research. Molecular biology provides new opportunities for the study of maize and other plants. This article reviews the physiological and biochemical responses of maize to high temperatures and drought, low temperatures, heavy metals, salinization, and diseases, as well as the molecular mechanisms associated with them. Special attention is given to key transcription factors in signal transduction pathways and their roles in regulating maize stress adaptability. In addition, the application of transcriptomics, genome-wide association studies (GWAS), and QTL technology provides new strategies for the identification of molecular markers and genes for maize-stress-resistance traits. Crop genetic improvements through gene editing technologies such as the CRISPR/Cas system provide a new avenue for the development of new stress-resistant varieties. These studies not only help to understand the molecular basis of maize stress responses but also provide important scientific evidence for improving crop tolerance through molecular biological methods.

## 1. Introduction

Maize (*Zea mays* L.) is an important food, economic, and fodder crop that is widely cultivated around the world. According to data from Cngrain, the global output of maize in 2023 exceeded 1220.794 million tons, an increase of nearly 1000 million tons compared to 2019 (www.cngrain.com, accessed on 6 November 2024). According to data from China’s National Bureau of Statistics, the cultivated area of maize reached 44,218.90 thousand hectares in 2023, and production reached 288.8423 million tons, accounting for 41% of the total grain output (Information from the National Bureau of Statistics of China: https://data.stats.gov.cn/easyquery.htm?cn=C01, accessed on 6 November 2024). Maize holds a very important position in China’s national economy and people’s lives. The production of maize far exceeds that of rice, making it the largest grain crop in China. The growth and development of maize is highly dependent on suitable climatic and soil conditions [1,2,3]. However, against the backdrop of global climate change and increasing human production and activities, the transformation of farming systems, the over-simplification of cultivated varieties, and the gradual increase in cultivated areas have brought great challenges to maize production. Adverse environmental stress factors such as drought, low temperatures, waterlogging, salinization, diseases, and heavy metals are the main limiting factors affecting maize growth and seriously affecting maize yields.

The growth and development process of maize is essentially the plant’s continuous adaptation and resistance to changes in the external environment. Maize faces numerous abiotic stresses throughout its life cycle, leading to extensive changes in its morphology, physiology, and cellular processes. The combination of different stresses further exacerbates these challenges [4].

The ability of plants like maize to resist and endure adverse conditions is known as plant stress resistance or simply resistance hardiness. This capability is gradually formed through the plant’s continuous coordination and adaptation to the environment over a long period. Unlike animals, the growth location of plants like maize is essentially fixed, and they cannot respond to changes in the external environment using their own locomotive functions and nervous systems as animals do. Therefore, plants like maize are often subject to the harm caused by adverse environments. However, through a long process of evolution and adaptation, maize can make full use of various methods to adapt to adversity in order to achieve its own survival and development. Maize’s adaptation to environmental stress relies on the activation of molecular network cascades involved in stress signal perceptions, signal transductions, and the expression of specific stress-responsive genes. The response of maize to abiotic stress involves an extremely complex regulatory network, including perception, transmission, and response to stress. When maize is subjected to stress, stress signals are perceived by various receptors within plant cells. Stress signals are then further transmitted downstream through secondary messengers. In this process, downstream stress-related transcription factors are activated through the mediation of protein modifications, thereby regulating the response of some stress-related genes, specifically through complex regulatory mechanisms, producing functional proteins that ultimately affect the plant’s tolerance to stress [5]. Ultimately, the study of these stress resistance pathways needs to be completed through molecular biology; hence, researching the stress resistance of maize, especially enhancing its stress resistance through molecular biology methods, holds significant theoretical and practical importance. The application of molecular biology in maize stress resistance research is a broad and in-depth field aimed at elucidating the physiological response mechanisms of maize under stress conditions, mining, and utilizing key genetic resources to enhance maize stress resistance, thereby improving the maize yield and quality. The application of molecular biology in maize stress resistance research is extensive and profound, and these applications not only help to reveal the physiological and molecular mechanisms of maize under stress conditions but also promote the breeding of stress-resistant maize varieties. The maize varieties produced using molecular biology techniques are diverse and often improved through gene cloning, transgenic technology, gene editing, and other means to enhance their stress resistance, yield, quality, and other characteristics. Understanding the molecular and genetic basis of maize’s perception and response to naturally occurring abiotic stresses is of great significance for improving crop tolerance through molecular biology methods.

This paper reviews the current understanding of the selection of stress-resistant genes in maize plants under multiple stresses, the pathways of stress signal perception and transduction, stress-resistance-related metabolic pathways, the genetic dissection of stress-resistant traits, and the application of genomics and bioinformatics in maize stress resistance research. Understanding the molecular and genetic basis of plants’ perception and response to naturally occurring abiotic stresses is of great significance for improving crop tolerance through molecular biology methods, and it also provides opportunities for the development of stress-resistant maize varieties.

## 2. Molecular Mechanisms of Maize Stress Resistance

During their growth and development, plants often face continuous or simultaneous threats from various biotic and abiotic stresses, which severely disrupt their physiological and metabolic processes. In recent years, significant progress has been made in the study of maize stress resistance. Researchers have used genomics, transcriptomics, and proteomics to uncover and analyze a wealth of genetic resources related to maize stress resistance. It has been discovered that throughout the long process of evolution, plants have developed a series of complex mechanisms to respond to stress, capable of rapidly sensing, processing, and transforming stress signals. Plants adapt to various environmental stimuli by altering their physiological metabolism. The plant stress response mechanism involves multiple components, and a timely and effective signal transduction system is essential for plants to respond quickly to stress. Moreover, transcription factors, acting as key molecular switches in stress signal transduction pathways, have also become a focal point in the study of maize stress resistance.

### 2.1. Analysis of Maize Stress Resistance-Physiological and Biochemical-Mechanisms

Stress constraints severely limit the growth, development, planting area, yield, and quality of maize. Under stress conditions, not only do morphological changes occur in maize plant. Its internal ecology, such as a series of physiological and biochemical processes within the cells are also significantly affected. These include the state of biomembranes, reactive oxygen species homeostasis, enzyme activities, osmotic regulatory substance content, hormone content, the induction of stress protein expression, and cross-adaptation to different stresses [6,7,8,9].

#### 2.1.1. Physiological and Biochemical Mechanisms of Maize Stress Resistance Under High-Temperature and Drought Conditions

Maize experiences varying degrees of impact on growth and development under high-temperature and drought stress at different growth stages. Seedlings become weak, and young plants develop poorly. After exposure to high temperatures during the flowering period, the fertilization and setting rate decrease. High temperatures during the grain-filling stage accelerate the growth process, leading to reduced grain weight. High temperatures and drought cause a yield reduction at all stages of maize growth because maize needs to escape high-temperature and drought stress by accelerating growth. In addition, it maintains a rich water content to avoid damage, and finally, it enters a drought-resistant defense state [10]. To make more efficient use of water, maize has multiple phenotypic traits in its morphological structure to maintain physiological water stability within the plant. This can be achieved in the following ways: First, maize forms an efficient root system that immediately absorbs a large amount of water [11,12,13,14]. The root system responds to changes in soil moisture at both the cellular scale and throughout the entire root system architecture. The root stem cell niche, meristem, and vasculature each coordinate responses for drought. During water scarcity, the root system architecture undergoes morphological changes to enhance its ability to absorb both water and nutrients. Under drought conditions, resources are protected through crown root senescence. Subsequently, a temporary halt in the growth of lateral roots is caused. Maize roots under water deficit stress exhibit enhanced growth that shifts a greater amount of exploration deeper in the soil column [15,16]. These modifications can be traced to coordinated cell division, elongation, and differentiation events in the root apex. Second, the stomata on the leaves, composed of two guard cells, regulate the exchange of gases and water and play a significant role in response to high-temperature and drought stress [17]. Maize improves its drought resistance by controlling stomatal closure to reduce water loss and can also enhance drought resistance by reducing stomatal density [18,19,20]. This helps to cope with high-temperature and drought conditions [21,22]. Third, there is osmotic adjustment within plant tissues. Maintaining cellular water balance by increasing the content of osmotic regulatory substances to alleviate the damage of environmental stress on cells.

Activated stress response pathways include phytohormone signaling, antioxidant and metabolite production, and mobilization. Plant hormone signaling pathways, such as abscisic acid (ABA) and salicylic acid (SA), play crucial roles in stress responses. Antioxidants, which include both enzymatic and non-enzymatic antioxidant systems, are vital for combating oxidative stress. Key antioxidant enzymes include superoxide dismutase (SOD), catalase (CAT), and peroxidase (POD), which work together to scavenge reactive oxygen species (ROS) and protect plants from oxidative damage. The production and mobilization of metabolites are also important; plants mobilize stored carbohydrates and lipids to maintain an energy supply and cellular functions. For instance, under stress, plants may increase the levels of soluble sugars and proteins to cope with adversity [23]. Maize escapes drought stress by accelerating growth, followed by maintaining a rich water content to avoid damage, and finally enters a drought-resistant defense state.

#### 2.1.2. Maize Stress Resistance Physiological and Biochemical Mechanisms Under Low Temperature

As global climate change progresses, a variety of extreme weather events occur, and in addition to drought, low temperatures causing freeze and cold damage also severely affect the growth and yield of maize. Low temperatures can lead to the formation of ice crystals, which can cause irreversible damage to the cells of plants such as maize. Low temperature-induced intercellular freezing causes severe dehydration of the maize cell protoplasm, leading to protein denaturation or irreversible gelation of the protoplasm; the mechanical pressure of ice crystals can also destroy the protoplasmic cells, causing mechanical damage; if the temperature suddenly rises, the ice crystals melt rapidly, and the protoplasm cannot absorb water and expand in time, leading to tearing damage and damage to the cell membrane permeability, causing an imbalance of ions inside and outside the cell, and resulting in increased conductivity. Therefore, the relative extrusion rate of electrolytes has become an important indicator for identifying plant cold tolerance. Besides intercellular freezing, intracellular freezing damages the plasma membrane, organelles, and the entire cell, causing fatal damage and ultimately leading to a decrease in maize yield [24]. Therefore, the structure of the cell membrane is most closely related to the cold resistance of maize. Maize varieties with strong cold resistance have a slower increase in membrane permeability, while varieties with weak cold resistance have a rapid increase in membrane permeability. Under low temperatures, biological membranes can shrink, leading to the formation of channels or cracks, which causes changes in the membrane’s morphology. Additionally, the activity of ATPase on the membrane can decrease, weakening the active transport capacity and allowing sugars and potassium ions (K+) to leak out of the cell. Research on the changes in chloroplast membrane phospholipids under low-temperature stress found that the lower the temperature, the greater the damage to membrane phospholipids. The biosynthesis of phospholipids is related to cold resistance, possibly because an increase in polar lipids lowers the phase transition temperature of membrane lipids. Therefore, it can be inferred that an increase in membrane phospholipids can enhance the cold resistance of plants. Studies have shown that phospholipase D (PLD) plays a particularly important role in the cold resistance response of maize, as it can hydrolyze membrane phospholipids to produce phosphatidic acid (PA), which acts as a signaling molecule involved in regulating plant responses to low temperatures. Specifically, PA enhances the plant’s tolerance to freezing stress by affecting the stability of the cell membrane and regulating the expression of genes associated with cold response. Therefore, it can be considered that under cold conditions, PLD and its hydrolysis product, phosphatidic acid, play a significant role in the types of phospholipids synthesized by maize [25]. Free proline, as an ideal osmotic regulatory substance, can promote the hydration of proteins, giving plants a certain degree of resistance and protective effect when they are under low-temperature stress [26]. Studies have shown that when maize is subjected to low-temperature stress, the content of proline in the maize increases significantly, and the accumulation of proline in maize increases with the decrease in temperature at any stage of the entire growth and development process [27]. Soluble sugars (including glucose, sucrose, fructose, and galactose), which also act as osmoregulatory substances, can improve cell osmotic potential, provide energy and substrate, induce cold resistance physiological and biochemical processes, stabilize the cell membrane and enzymes, and act as protective agents under low-temperature stress in maize.

#### 2.1.3. Physiological and Biochemical Mechanisms of Maize Stress Resistance in Heavy Metal-Rich Environments

Heavy metals are divided into essential elements and non-essential elements for plant growth. Essential elements such as copper (Cu), zinc (Zn), nickel (Ni), and cobalt (Co) are essential or beneficial mineral nutrients for plant metabolism and growth but can become toxic to plants when their accumulated concentrations are too high. Other trace metals or metalloids such as cadmium (Cd), mercury (Hg), arsenic (As), lead (Pb), and chromium (Cr) are non-essential elements and are generally highly toxic to plants, even at relatively low concentrations. Maize plants suffering from heavy metal toxicity often exhibit stunted growth, decreased leaf area, leaf chlorosis and/or necrosis, and turgor loss [28,29]. Maize plants have evolved different mechanisms to cope with heavy metal stress. Resistance or tolerance to heavy metals is manifested through exclusion mechanisms, i.e., extracellular mechanisms that limit metal uptake into plant cells and detoxification mechanisms within the cell that sequester metals in the vacuoles and extrude metals out of the cells. Cell-wall binding with heavy metals may reduce their uptake into the cells, thus constituting an exclusion mechanism. Other mechanisms include complexation (the majority of trace metal ions inside plant cells are bound to low-molecular-weight ligands and peptides/proteins), vacuolar sequestration (in mature plant cells, the vacuole is the largest organelle for the storage of ions and metabolites; tonoplast transporters play a key role in trace metal sequestration in the vacuoles), efflux (the export of trace metals out of the root cells is an important detoxification mechanism in plants), and translocation to the above-ground tissues (translocation of toxic trace metals from roots to shoots can also be considered as a detoxification pathway to reduce trace metal toxicity to plant roots, if trace metals can be detoxified more efficiently in the shoots). In addition, the signal transduction pathway plays a major role in maize in response to heavy metal stress [30].

#### 2.1.4. Physiological and Biochemical Mechanisms of Maize Stress Resistance Under Salt Stress

Reports indicate that, aside from halophilic bacteria, all other organisms are not tolerant to salt at the molecular level. Maize is a sweet soil plant, and its salt tolerance is lower compared to many other plants; it is moderately sensitive to salt [31]. When subjected to salt stress, maize often fails to grow normally, leading to incomplete seedling development and reduced fruit, severely affecting yield. Salt stress can cause various physiological injuries to plants, and the three commonly recognized types are osmotic stress, ionic stress, and oxidative stress [32]. Osmotic stress occurs in the early stages of salt-alkaline stress, and its mechanism involves increasing soil osmotic potential and decreasing soil water potential, resulting in difficulty for plants in water transport. The rapid decrease in growth exhibited by plants under osmotic stress is similar to the initial response to water stress. The ionic stress phase reflects the genotypic differences of plants. The combined action of osmotic and ionic stress can lead to secondary oxidative stress in plants [33]. The ions Na^+^, Cl^−^, and SO_4_^2−^ from salt bring ionic stress to plants, along with secondary stresses such as osmotic stress, nutritional imbalance, and oxidative stress. Understanding the mechanisms of plant salt tolerance will be beneficial for improving crop salt tolerance or developing genetically engineered salt-tolerant crops.

#### 2.1.5. Physiological and Biochemical Mechanisms of Maize Stress Resistance Under Pathogen Stress

The immune system of maize against pathogenic bacteria is primarily composed of two branches. The first branch is the immune response triggered by pathogen-associated molecular patterns (PAMPs), known as PAMP-triggered immunity (PTI). This process is generally initiated by pattern recognition receptors (PRRs) on the plant cell membrane recognizing PAMPs—certain conserved structures that are easily exposed when pathogens invade, such as bacterial flagellin and fungal cell wall chitin. Plants perceive the presence of pathogens through a multitude of PRRs on their cell membranes. These PRRs are typically receptor-like kinases (RLKs), which consist of an extracellular recognition domain and an intracellular kinase domain. Upon recognizing PAMPs, PRRs activate signal transduction pathways through the interaction of receptor-like cytoplasmic kinases (RLCKs) within the cell membrane, such as the mitogen-activated protein kinase (MAPK) pathway, transmitting signals to the downstream cell nucleus. Alternatively, they may use the calcium-dependent protein kinases (CDPKs) pathway to relay signals back to the cell membrane, thereby activating a series of plant disease resistance responses [34]. The expression of plant resistance genes allows cells to respond to the invasion of pathogens, such as by producing antimicrobial compounds and thickening cell walls. If the PTI response is rapid and strong enough, it can combat invading pathogens, and the plant will exhibit disease resistance. The second branch is triggered by effector-triggered susceptibility (ETS), where plants, in their long-term competition with pathogens, have evolved a second branch of the immune system to counter ETS. This involves using specific proteins to monitor effectors (which are produced in large quantities by pathogens during invasion) and activate an immune response known as effector-triggered immunity (ETI). Through mutation and natural selection, plants have developed and retained genes corresponding to effectors. These genes encode proteins that, upon recognizing an effector, reactivate the disease resistance pathway through ETI. In research, these genes that recognize and combat effectors are referred to as resistance genes (R genes), because it is only when these R genes are present that plants have the ability to resist pathogens carrying corresponding effectors. The recognized effectors are known as Avirulence (Avr) proteins [35].

When facing stressors such as high temperatures and drought, low temperature, heavy metals, salinization, and diseases, maize has developed various strategies to cope through long-term evolution, involving plant cell walls, cell membranes, vacuoles, and other tissues. Osmotic regulation, ion regulation, and oxidative regulation are key, along with the synthesis and accumulation of organic compatible osmotic products, antioxidant enzymes, and metabolites as auxiliary measures. These are crucial at the physiological level for maize’s stress resistance [36].

### 2.2. Research on the Signal Transduction Pathways and Key Genes of Maize Under Stress

**Figure 1:**
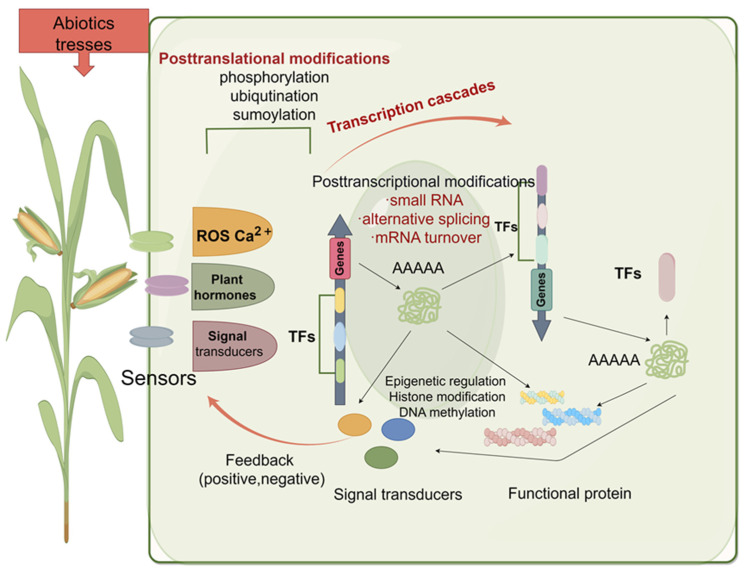
Maize abiotic stress resistance processes.

The above text describes how maize responds physiologically and biochemically to various adverse environmental conditions. It is observed that regardless of the type of stress maize encounters, the cell wall, cell membrane, and vacuoles are all involved, and osmotic, ionic, and oxidative regulations are indispensable. However, all complex physiological tolerance mechanisms rely on gene regulation at the molecular level. That is, plants can activate a series of genes involved in signal transduction and metabolic pathway regulation, reducing plant damage through the transcriptional activation and integration of effector genes mediated by transcription factors. In recent years, extensive research has been conducted on the stress resistance mechanisms of maize, and it has been found that when stress perception proteins within maize sense stress signals, they transmit these adverse signals within the plant. Generally, stress signals are thought to be initially perceived by membrane-bound proteins, which then stimulate downstream protein kinases and induce the expression of transcription factors (TFs) [37]. Secondary messengers, such as intracellular calcium ions (Ca^2+^) and reactive oxygen species (ROS), play an important role in the cellular signaling network responding to abiotic stress [38]. Different abiotic stresses often lead to similar secondary effects, including osmotic, ionic, and oxidative stress, triggering plant hormones, compatible osmolytes (small organic osmolytes, such as soluble sugars, polyols, amino acids, and their derivatives), and antioxidant enzymes (a group of proteins responsible for converting reactive oxygen into active molecules), protecting the stability of membranes, proteins, and cells [37]. Therefore, plants’ adaptation to environmental stress depends on the activation of molecular network cascades involved in stress signal perception, signal transduction, and the expression of specific stress-responsive genes (Figure 1).

Plants such as maize adapt and respond to various stresses through a series of complex signal transduction pathways. Plant hormones may act as initiating factors for the expression of stress-resistant genes. Stress can affect the physiological processes of plants by influencing the levels and activities of their own hormones, and plant hormones are key signaling molecules that regulate plant growth and development as well as adaptation to various biotic and abiotic stresses [39,40]. This leads to the formation of signal transduction pathways in plants like maize, which can be divided into two types: one that depends on the stress hormone ABA and another that does not depend on ABA. The ABA-independent signal transduction pathways can rely on signaling by jasmonic acid (JA), salicylic acid (SA), ethylene (ET), gibberellins (GA), brassinosteroid (BR), cytokinins (CKs), and strigolactones (SL), among others. In the stress resistance of maize, the ABA signal transduction pathway plays a significant role and is central to the response to abiotic stresses [41,42]. However, the function of ABA must be precisely regulated to prevent sustained reactions from causing damage to the plant.

#### 2.2.1. Research on Signal Transduction Pathways and Key Genes of Maize Under High-Temperature and Drought Stress

Under high-temperature and drought conditions, the physiological and biochemical mechanisms of maize stress resistance regulate the signal transduction process in response to drought, which includes ABA-dependent and ABA-independent pathways. For the ABA-dependent signal transduction process, dehydration signals stimulate local production of ABA in different plant organs. However, ABA production is found to be most efficient in the leaf mesophyll cells compared to the root tissues [43]. When ABA accumulates to a certain level, it stimulates downstream signaling and ABA functions in conjunction with other pathways.

ABA is one of the most important stress hormones in plants, and its active regulatory role under drought stress is widely recognized. ABA can promote plant stomatal closure, regulate root growth, enhance drought resistance, and more [44]. BRs: BRs are a class of plant sterol hormones that participate in plant growth and development as well as in responses to stress. BRs can enhance plant stress resistance, including drought stress [45]. GA: GA promotes plant growth and development. Under drought stress, GA is also involved in regulating plant growth and development processes. ET: It plays an important role in plant responses to stress, including drought stress. Ethylene can regulate plant growth, flowering, fruit ripening, and other processes [46] JA: JA plays a key role in plant defense responses. Under drought stress, JA can regulate plant antioxidant capacities and defense enzyme activities and help plants cope with stress challenges [47]. These plant hormones collectively participate in regulating the physiological and biochemical processes of plants under drought stress, helping plants adapt to and cope with adverse environmental conditions. In the ABA-dependent stress signal transduction pathway, key transcription factors that depend on ABA include the SnRK 2, ABI 5, bZIP, B3 HD-ZIP, GRAM, and the WRKY family [48]. In the ABA-independent pathway, key transcription factors include the DREB family, ZFHD family, and HSF family. However, transcription factors such as AP2/ERF, ABF, MYC, MYB, and NAC also function in both ABA-dependent and ABA-independent signaling pathways [49,50]. For example, some NAC transcription factors can also regulate plant stress responses by modulating the signaling pathways of other plant hormones, such as the ET and JA pathways [44]. These transcription factors interact within various signaling pathways to exert their effects, and the genes they regulate also play roles in these pathways. This demonstrates that stress resistance in plants such as maize is part of a complex and interactive network system, necessitating further exploration of the interactions between individual genes, proteins, and signaling pathways.

Identification of components and genes in the core ABA signal transduction pathway has been conducted. ABA is an important plant hormone that perceives and responds to environmental stresses by binding to PYR/PYL/RCAR family proteins. PYLs (members of the PYR/PYL/RCAR family) act as ABA receptors and function at the top of the negative regulatory pathway by inhibiting PP2Cs (type 2C protein phosphatases) to control ABA signaling. PYLs interact with PP2Cs to form a double-negative regulatory system of PYR/PYL/RCAR-PP2C-SnRK2, which regulates the ABA signal transduction pathway and its downstream responses. PYLs suppress the activity of SnRK2 protein kinases through their interaction with PP2Cs. PP2Cs physically interact to neutralize SnRK2s, rendering them inactive and thus inhibiting the activation of transcription factors that mediate the expression of ABA-responsive genes. In the presence of ABA, the PP2C interacts with the ABA receptor RCAR/PYR/PYL, undergoes a conformational change that allows it to interact with PP2C and activates SnRK2, thereby phosphorylating downstream substrates to mediate stress responses. In the absence of ABA, PP2C inhibits SnRK2s through physical interaction, and in the presence of ABA, the conformational change in PP2C upon binding to the ABA receptor releases SnRK2s, allowing their activation. This indicates that ABA regulates plant responses to adversity through the PYLs-PP2Cs-SnRK2s pathway [51]. The activated SnRK2s promote stomatal closure by phosphorylating ion channels such as KAT1, SLAC1, and SLAH3, induce an ABA-responsive gene expression by phosphorylating transcription factors such as ABA-responsive element-binding factors (ABFs), and may regulate many other processes through phosphorylating other putative substrates [52,53]. Through the yeast two-hybrid assay, studies involving ABA have discovered its role in stress resistance [54]. Among them, the homolog of *Arabidopsis* (*Arabidopsis thaliana*) OPEN STOMATA 1 (OST1)/SnRK2.6, ZmOST1, is accumulated after drought and ABA treatment. The absence of ZmOST1 impairs ABA-regulated stomatal movement and resistance. Under drought conditions, ZmPP2CA10 participates in ABA signal transduction by interacting with SnRK2s and ZmPYL ABA receptors [55]. In the study by Wang, Y.G. et al., it was discovered that ZmPYLs, serving as ABA receptors, do not uniformly exhibit upregulation in gene expression under ABA treatment. An evaluation of the gene expression patterns of *ZmPYLs* and *ZmSnRK2s* in the ABA-treated maize inbred line B73 was conducted. Among these, the expression of *ZmPYL2* was downregulated, while the expression of *ZmPYL1/4/5/6/7/11/13* was upregulated. After ABA treatment, the expression levels of *ZmSnRK2s* in the roots of the maize inbred line ‘B73’ were all upregulated. In Arabidopsis, there are differential interactions between PYLs and PP2Cs [56]. To analyze the redundancy and diversity of interactions between ABA receptors and coreceptors, 13 *ZmPYLs* and 16 *ZmPP2Cs* were cloned from the inbred line B73, and the yeast two-hybrid method was used to detect over 260 possible interactions among them. However, the specific modes of cooperation have not been deeply investigated and warrant further scientific exploration [54]. Other substances involved in ABA signal transduction that participate in plant stress resistance processes include the S-type anion channel ZmSLAC1 mediates stomatal closure during the drought response in maize. The plasma membrane (PM)-localized Ca^2+^-dependent protein kinases (CPKs) ZmCPK35 and ZmCPK37 interact with and activate ZmSLAC1, inducing stomatal closure signaling to ABA and Ca^2+^, and the PM-localized ZmRBOHC, promotes ROS accumulation in guard cells, thereby inducing stomatal closure [57]. The mitogen-activated protein kinase (MAPK) cascade plays an important role in the drought response [58]. For example, ZmPP84, a member of the PP2C branch F, interacts with and dephosphorylates a member of the MAPKK family, de ZmMEK1, thereby inhibiting its activity. After ABA accumulation, ZmPP84 releases the inhibitory effect on ZmMEK1, and ZmMEK1 enhances the activity of salt-induced mitogen-activated protein kinase 1 (ZmSIMK1), which mediates ZmSLAC1 phosphorylation and induces stomatal closure. From completing the ABA signal transduction to the plant’s response to stress [59].

Most stress responses involve the ABA pathway, but there are also stress response pathways that are independent of ABA. For instance, under high-temperature and drought stress, maize can utilize the biosynthesis and signal transduction of ethylene to respond, which can lead to changes in maize yield [60]. Under drought stress, the knockout of the gene encoding 1-aminocyclopropane-1-carboxylate synthase 6 (ACS6), which synthesizes Acetyl-CoA Carboxylase (ACC)\(the precursor of ethylene), improves grain yield compared to the wild type [61]. The overexpression of auxin-regulated genes such as *ZmARGOS1*/*ZeamaysARGOS1* (*ZAR1*) and *ZmARGOS8* reduces the plant’s sensitivity to ethylene, thereby enhancing drought resistance and increasing grain yield under both normal and drought conditions [62]. Furthermore, editing the promoter sequence of ZmARGOS8 can upregulate the expression of *ZmARGOS8*, thereby reducing the sensitivity of maize to ethylene, enhancing its drought tolerance, and increasing its yield under conditions of water scarcity [63].

#### 2.2.2. Research on Signal Transduction Pathways and Key Genes of Maize Under Low-Temperature Stress

Plant responses to low temperatures are highly complex and are regulated by the crosstalk of various mechanisms such as cold signaling, ABA signaling, BR signaling, and photoperiodic responses [64]. Cold stress greatly affects plant metabolism and transcription levels. Most of the effects on plant metabolism stem from the direct inhibition of metabolic enzyme activity and reprogramming of gene expression at low temperatures [65]. The C-repeat binding factor C-repeat binding factor/Dehydration-responsive element binding factor (CBF/DREB1)-centered C-repeat binding factor—cold-regulated gene (CBF-COR) pathway is an important signal transduction pathway for maize to adapt to low temperatures. Cold stress rapidly induces the expression of many transcription factors, ICE1 (Inducer of CBF expression), is a MYC-like bHLH transcription factor that can participate in the expression of CBF3/DREB1A under low-temperature conditions and can also regulate most transcription factors and COR genes (Cold Regulated Genes) associated with cold regulation. Studies have shown that ICE1 is simultaneously subjected to SUMOylation and polyubiquitination modifications, followed by proteasomal degradation. SUMOylation is mediated by the SUMO E3 ligase SIZ1, and polyubiquitination is mediated by the ubiquitin E3 ligase HOS1. These two modifications are related to the stability of ICE1 and further affect the expression of plant cold stress genes, as ICE1 can precisely regulate CBFs (C-repeat binding factors), which can activate the expression of many cold-regulated protein genes [65]. In addition, the cold stress induction of CBF and COR genes is controlled by the circadian clock [66]. In the cold resistance of maize, the positive regulator SnRK2.6 in the ABA (abscisic acid) signaling pathway also plays a key role. SnRK2.6 is a protein kinase that plays a key role in plant stress responses and is involved in regulating stomatal opening and closing in response to environmental changes [67]. In the ABA signal transduction pathway, the activation of SnRK2.6 is achieved through phosphorylation, including the phosphorylation of Ser171 and Ser175 sites on its activation loop. These phosphorylation events are key steps in the activation of SnRK2.6. Studies have shown that cold stress can activate SnRK2.6/OST1, and SnRK2.6 interacts with ICE1 and phosphorylates it, thereby activating the CBF-COR gene expression system and enhancing freezing tolerance [68].This also confirms the cross-regulation among various signaling pathways. However, based on current research on maize cold stress resistance, more research is needed by scientists on the cross-actions of various signal transduction pathways.

#### 2.2.3. Research on Signal Transduction Pathways and Key Genes of Maize in Heavy Metal Environments

Like most plants, maize primarily uses ABA signaling to counteract the adverse effects of heavy metal stress. ABA can improve the physiological processes, antioxidant defense systems, and accumulation of osmolytes in maize, thereby reducing the impact of heavy metal stress on the plant [69]. Research indicates that the maize transcription factor *ZmbHLH105* directly binds to the promoter regions of two key ABA biosynthesis genes, *ZmNCED1/2*, activating their transcription and thus increasing ABA levels and Cd tolerance. Exogenous ABA supplementation increases the lignin content in the roots of maize under Cd stress, and the overexpression of *ZmbHLH105* promotes lignin synthesis, while ZmbHLH105-RNAi weakens this effect. This suggests that ZmbHLH105-mediated Cd tolerance may involve ABA-induced lignin deposition and the thickening of the cell wall. Furthermore, the expression of genes related to Cd transport is inhibited in ZmbHLH105-overexpressing lines [70]. Additionally, ATPases, as enzymes involved in ATP hydrolysis, may affect the intracellular ATP/ADP ratio, which in turn could influence ABA signaling and biological effects. In maize, the ATPase (HMA) ZmHMA, a member of the HMA family proteins, can positively regulate tolerance to Cd and Zn [71].

Furthermore, the addition of exogenous JA (jasmonic acid), GA (gibberellins), and SA (salicylic acid) can enhance tolerance to stress caused by Cd, As, and Pb. For instance, the allene oxide cyclase (AOC) gene in maize, which is a key enzyme in the biosynthesis of JA, is regulated by the expression of various hormones, including JA itself, SA, GA, and ABA. This suggests that in maize, JA, GA, SA, and ABA might work together to influence the response to heavy metal stress through mutual regulation [72].

#### 2.2.4. Research on Signal Transduction Pathways and Key Genes of Maize Under Salt Stress

Plant hormone signaling pathways control the adaptation process of plants under salt stress. The positive correlation between the degree of inhibition of leaf growth and the ABA content in maize leaves under salt stress suggests that ABA signaling plays an important role in plant salt stress adaptation [73]. Under salt stress conditions, the expression of genes related to terpenoid synthesis in maize changes, including the expression of terpene synthase genes (TPS2, TPS3) and key enzymes in terpenoid compounds such as GGPS4, which may play an important role in salt tolerance [74]. Secondly, ethylene is another important hormone involved in stress response in plants. After plants are subjected to salt stress, the content of ethylene and its precursor ACC will increase [75]. The application of ethylene to plants can enhance their salt tolerance [76]. After ethylene signaling is activated, the plant’s ability to clear ROS produced by salt stress significantly increases, thereby reducing the damage caused by ROS to plants [77].

BR signaling acts as a positive regulator in response to salt stress. In rice, the loss of function of the negative regulator of BR signaling, BIN2, enhances salt tolerance, and increasing endogenous BR content through RNA interference also improves salt tolerance. In maize, genetic engineering approaches to increase the levels of brassinosteroids within the plant may enhance tolerance to salt stress. For example, by constructing overexpression vectors, introducing genes related to brassinosteroid synthesis into maize, and then selecting and identifying transgenic lines, salt tolerance in maize can be improved. By combining genome-wide association analysis and linkage analysis for salt tolerance in maize, two excellent salt tolerance genes ZmSAGs (salt-associated genes) were identified: ZmSAG4 and ZmSAG6. ZmSAG4 encodes the SEC23 protein, which is mainly involved in the transport of proteins within the endoplasmic reticulum. The other gene, ZmSAG6, encodes the MRE11 protein, which is primarily involved in the DNA damage repair process of plants under salt stress. After overexpressing these two genes, it was found that the salt tolerance of the overexpressing strains was stronger than that of the wild type. Through candidate gene association analysis, seven single nucleotide polymorphisms (SNPs) significantly related to the salt stress response were located in ZmSAG4. If a haplotype Hap 4 possesses two of these excellent allelic variations simultaneously, it exhibits a stronger tolerance to salt stress [78]. Additionally, hormone signals such as auxin, jasmonic acid, and gibberellins have also been reported to play roles in plant salt stress responses [79].

#### 2.2.5. Research on Signal Transduction Pathways and Key Genes of Maize Under Pathogen Stress

Systemic Acquired Resistance (SAR) in plants is a defense response mediated by salicylic acid (SA) that enhances resistance to a broad spectrum of pathogens. It is a defensive response initiated after plants have been infected by pathogens. This response begins with the recognition between plant resistance genes (R) and avirulence genes (avr) of pathogens, and the defense signal is passed down through SA [80]. Among the key regulatory factors is NPR1 (Non-expression of Pathogenesis-Related Protein 1), whose activation induces the expression of pathogenesis-related (PR) protein genes [81,82]. Additionally, in maize disease resistance, ABA may also regulate immune responses through interactions with other hormones and signaling molecules. ABA can interact with other plant hormones, such as SA and JA, to collectively modulate the plant’s defense against pathogens [83].

### 2.3. Metabolic Pathways and Key Genes Related to Maize Stress Resistance

There are changes in the metabolic pathways of maize under stress conditions, especially those closely related to stress resistance (such as sugar metabolism and fatty acid metabolism). By regulating key genes or enzymes in these metabolic pathways, the stress resistance of maize can be improved. To gain a deeper understanding of the metabolic pathways involved in maize stress resistance, physiological and biochemical indicators can be measured (such as measuring the content of soluble proteins, antioxidant enzyme activity, and endogenous hormone content in maize under stress conditions). On the basis of transcriptomics and whole-genome analysis, differentially expressed genes (DEGs) can be identified and further explored for their functions and metabolic pathways in stress response through GO enrichment analysis and KEGG pathway analysis. After identifying the stress-resistant metabolic pathways, key genes on the pathways can be selected for gene cloning and functional validation, gene expression analysis, association analysis, and molecular markers, and transgenic technology can be used to quantitatively analyze their expression under different stress conditions and measure physiological and biochemical indicators to determine whether these genes are involved in the regulation of stress resistance.

It was found that transcription factors resist low temperatures by regulating amino acid metabolism [84]. Energy metabolism is also involved in maize stress resistance: under low-temperature stress, the metabolic regulatory network of maize involves energy metabolic pathways, which may be related to maintaining the balance of intracellular energy. By annotating DEGs from RNA-seq data with KEGG, only the cold-tolerant maize inbred line HA663 had 19 DEGs annotated to starch and sucrose metabolism under low-temperature stress for 24 h. Lipid metabolism: The lipid metabolic pathways of maize, including the biosynthesis of fatty acids and triglycerides, are important for increasing the content of oil and fatty acids and may be related to stress resistance. The biosynthesis of secondary metabolites: When maize responds to stress, it may activate the biosynthetic pathways of certain secondary metabolites, such as the biosynthesis of phenylpropanoids, which may be related to the plant’s defense mechanisms [85]. Plant hormone signal transduction: Plant hormones such as abscisic acid, salicylic acid, and jasmonic acid play a key role in plant stress responses, and their signal transduction pathways are an important part of maize stress resistance. MAPK signal transduction pathways: These pathways play a central role in signal transduction in plants under stress. Carbohydrate metabolism: When maize faces stress, its carbohydrate metabolic pathways may change to maintain growth and development. Studies have shown that signal pathways responding to drought, high salinity, and low-temperature stress can be divided into three types [86]. The first type is the osmotic stress/oxidative stress signal pathway, where mitogen-activated protein kinase (MAPK) plays an important role. This pathway generates antioxidants and soluble osmotic adjustment substances to regulate the cell cycle, thereby protecting and repairing various damage caused by stress to cells. The second type is the signal pathway that depends on the activation of LEA-like (Late Embryogenesis Abundant) genes by Ca^2+^. The third type represents the Ca^2+^SOS signal pathway, which regulates the internal ion balance of cells to transmit stress signals. Among these, the MAPK metabolic pathway plays an important role under drought, high salinity, and low-temperature stress. MAPK is mainly composed of three types of kinases, namely mitogen-activated protein kinase kinase kinase (MAPKKK), mitogen-activated protein kinase kinase (MAPKK), and mitogen-activated protein kinase (MAPK), which connect upstream and downstream regulatory factors through phosphorylation [87,88]. In the MAPK cascade signal, MAPKKK is the most upstream, activated by the upstream activating signal, and phosphorylates the downstream MAPKK. The activated MAPKK phosphorylates the downstream MAPK. The activated MAPK can activate downstream proteins within the cell, phosphorylating various substrates, including transcription factors, protein kinases, and structural proteins, thereby activating cellular responses [89,90]. All MAPKKs have a MAPK docking domain at the N-terminus, K/R-K/R-K/R-X1-6-L-X L/V/I. In the MAPK cascade signal, MAPKK is in the middle position, can be phosphorylated by MAPKKK, and can also phosphorylate MAPK, thus forming multiple MAPK cascade signal pathways. MAPK is located at the most downstream protein kinase in the cascade reaction and is currently divided into four subfamilies, A-D. In maize, 19 *MAPK* genes have been found and are named *ZmMPKs* [91,92]. The functions of maize MAPK pathway genes have recently become a hot topic in maize stress resistance research. Under dual stress of drought and Cd, overexpression of maize ZmMAPKKK18 (Mitogen-Activated Protein Kinase Kinase Kinase) can improve the drought resistance of maize. Studies in other plants have found that MAPK can participate in drought resistance, salinity tolerance, disease resistance, and cold resistance. In addition, when maize faces abiotic and biotic stress, the regulation of the jasmonic acid-mediated signaling pathway, response to wounding, regulation of defense response, phenylpropanoid metabolic process, secondary metabolic process, lignin metabolic process, and phenylpropanoid biosynthesis pathway all play a role [93]. In the study of cold resistance of Russian-introduced maize, it was found that most of the differential genes related to cold resistance are enriched in starch and sucrose metabolism, fructose and mannose metabolism, brassinosteroid biosynthesis, tyrosine metabolism, nicotinate and nicotinamide metabolism, cyanoamino acid metabolism, glycerolipid metabolism, galactose metabolism, phenylpropanoid/anthocyanin biosynthesis pathway, plant-pathogen interaction, and glutathione and ascorbate conversion KEGG pathways to enhance stress resistance.

### 2.4. Application of Genomics and Bioinformatics in Maize Stress Resistance Research

The cloning of genes is the foundation for carrying out various functional studies. With the development of high-throughput sequencing methods, analytical techniques such as transcriptomics (RNA-seq), proteomics, quantitative trait locus (QTL) mapping, and genome-wide association studies (GWAS) are increasingly being used to mine stress-related genes in plants like maize to reveal the molecular mechanisms of plant stress resistance.

Transcriptomics and proteomics analyses are essential tools for studying plant responses to specific adverse environmental conditions. Through these analyses, scientists can directly observe changes in gene expression and protein abundance under stress, thereby revealing the physiological mechanisms involved in stress responses and identifying stress-responsive genes. Transcriptome analysis of maize embryos and leaf meristems under water stress has shown that the transcription abundance of a large number of cell division and cell cycle-related genes decreases, and the expression levels of starch and sucrose-related genes in ovaries and leaves change significantly, indicating that under water stress, maize can accelerate the synthesis and metabolism of carbohydrates to increase the plant’s drought resistance [94].Through RNA-seq analysis of the drought-resistant maize line RIL70 and the water-sensitive line RIL93, it was found that genes related to cell wall biosynthesis and transmembrane transport processes were highly expressed in the drought-resistant line. These genes may be involved in signal transduction, leading to accelerated metabolism in maize under drought conditions, thereby enhancing the drought resistance of the drought-resistant line RIL70 [95]. Transcriptome sequencing analysis of two maize inbred lines with different salt tolerances under salt stress conditions identified differentially expressed genes related to redox processes, biological regulation, and the plasma membrane while also revealing the molecular mechanism by which anthocyanins enhance the salt tolerance of maize seedlings [96]. In recent studies, salt treatment was applied to two extreme populations derived from the cross between AS5 and NX420, and combined with BAS-seq, 77 genes were identified. By integrating transcriptome and BAS-seq data, it was found that *Zm00001d053925* was detected in both AS5 and NX420, with its expression doubling under salt stress. The newly discovered gene *Zm00001d053925*, which significantly affects the salt tolerance of maize seedlings, provides important genetic resources for maize salt tolerance breeding and also offers a potential target gene for developing more salt-tolerant maize varieties [97]. In the study of the mechanisms by which maize seeds cope with salt stress using proteomics, it was found that proteins involved in seed storage, energy metabolism, stress response, and protein metabolism showed differential expression, with a particular focus on the increased expression of proteins responding to abscisic acid signals under salt stress [98]. In the study of cold resistance in maize, transcriptomics research has also identified genes related to circadian rhythms and cell membrane and cell wall systems that are associated with cold stress tolerance [99,100]. There are some cold signal transduction genes in maize, such as *ZmCDPK1*, *ZmMAPKKK*, and *ZmRR1*, which are related to plant cold tolerance [101]. In plants, a trait is often controlled by multiple genes, and the location where these multiple genes exist is referred to as QTL. Cloning of functional genes in plant QTLs was an extremely tedious and difficult process before the development of second-generation sequencing technology. However, with the continuous development of sequencing technology and the release of reference genomes for various species, cloning of target genes has become simpler. QTL mapping is an important strategy for locating stress-resistant genes using associated markers. In recent years, advanced and rapid sequencing methods have been able to obtain high-density genetic information, which allows for the discovery of a large number of molecular markers and fine mapping of related QTLs. This has been widely applied in the study of drought resistance, cold resistance, disease resistance, and heavy metal resistance in plants such as maize. In the QTL mapping related to maize drought resistance, Almeida and others identified 83 and 62 genetic regions associated with crop yield and anthesis-silking interval (ASI) in multiple environments and different genetic backgrounds [102]. Zhao and others identified 69 QTLs related to maize under drought stress, involving phenotypic traits such as plant height, ear height, ASI, ear weight, ear core weight, hundred-seed weight, and ear length [103]. In the study of maize cold resistance, QTL technology has demonstrated its advanced nature. Although maize originated in the tropics, its genetic characteristics include many that are related to cold resistance. Current research can utilize QTL to explore the genetic characteristics of maize’s cold resistance capabilities. Existing QTL mapping studies have revealed multiple gene loci associated with the activity of maize seedlings and basic physiological processes, such as photosynthesis. However, there is still a lack of in-depth understanding of the molecular mechanisms by which plants adapt to low-temperature environments [104,105]. By analyzing field data and transcriptome data of different maize inbred lines under low-temperature conditions over multiple years, researchers have identified three response mechanisms to low-temperature stress: photosynthetic structural mechanisms, cell wall protection mechanisms, and developmental process mechanisms. QTL mapping of maize recombinant inbred lines grown at temperatures below normal growth conditions for several years has revealed significant genetic regions associated with photosynthetic organ-related traits. This explains the involvement of photosynthetic mechanisms in the response process of maize to low-temperature stress [106]. Using QTL analysis to locate the cold resistance of cold-resistant and cold-sensitive materials, significant genetic regions associated with the growth and development of maize seedlings under cold stress at low temperatures were identified [107]. When a QTL analysis is conducted on a recombinant inbred line population derived from a cross between two parents with significantly different cold resistance, the resulting QTL loci are essentially consistent [108]. Studies have shown that QTL applications have achieved significant success in the genetic region localization of cold tolerance during the seedling stage of maize. However, the genetic mechanisms underlying different cold/freeze traits in different germplasms still require further research. The genetic mechanisms underlying different cold tolerance traits in different germplasms still require further research. QTLs are also widely applied in maize disease resistance, and an increasing number of researchers have identified a series of QTLs related to seedling blight, stalk rot, ear rot, cob rot, and seed decay (caused by *Fusarium subglutinans* infection, seed-borne *Fusarium subglutinans* infection) through resistance screening and combined with GWAS for identification and analysis [109,110,111,112].

The application of genome-wide association study (GWAS) has also facilitated the mapping of QTLs related to maize stress resistance, becoming an important method for identifying stress resistance in maize. In the study of maize drought resistance, this method was used to identify 83 drought-resistant genetic variations and to locate the drought-resistant gene *ZmVPP1* [113]. Combining GWAS with omics is also frequently used in maize stress resistance and is a powerful tool for fine mapping stress-resistant genes. By integrating GWAS data from 209 natural maize populations with drought transcriptome data, seven genes involved in the response to drought stress were identified [114]. Using GWAS (Genome-Wide Association Studies), researchers identified 43 SNPs (Single Nucleotide Polymorphisms) associated with 10 cold tolerance indicators in 125 maize inbred lines [115]. Another study discovered 19 significantly associated SNPs in 375 inbred lines, explaining 5.7% to 52.5% of the phenotypic variation in cold tolerance traits [116]. By combining GWAS with transcriptome analysis, researchers have identified 30 SNPs and 82 cold tolerance candidate genes in 222 natural maize populations, providing a molecular basis for understanding the genetic mechanisms of cold tolerance during germination in maize [117]. Under saline-alkali stress, GWAS analysis was conducted on nine traits of 200 natural maize populations, and in combination with transcriptome results, a total of five salt-alkali tolerance-related genes were identified, two of which are involved in the biosynthesis of flavonoids [110]. GWAS has limitations in improving the precision of trait gene localization, and to accurately locate trait genes, it is necessary to combine linkage mapping or transcriptomics. This method is commonly used in the localization of maize resistance genes. In a GWAS of 342 maize inbred lines, using mixed linear models and SNP association analysis, seven significant SNPs were identified, five of which on the sixth chromosome were associated with resistance to Fusarium stalk rot caused by Fusarium verticillioides, leading to the localization of the resistance gene [110]. In summary, transcriptomics, genomics, GWAS, and other technologies play an important role in the study of maize stress resistance. They can help researchers identify genes and molecular markers related to maize-stress-resistance traits, which can then be used for the genetic improvement of maize.

### 2.5. The Role of Transcription Factors in Maize Stress Resistance

**Figure 2:**
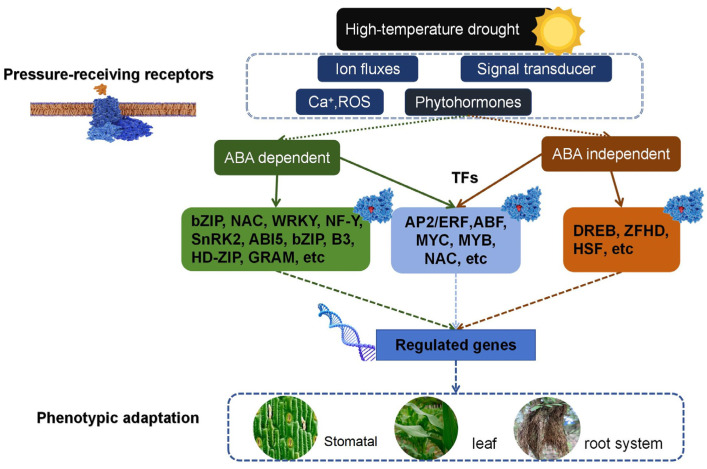
Transcriptional regulatory network.

Transcription factors are a group of proteins that can specifically bind to cis-acting elements in the promoter regions of eukaryotic genes. When plants sense external stresses such as drought, high salinity, and low temperature, transcription factors are activated, which in turn activate the expression of many stress-inducible genes, thereby enhancing the plant’s tolerance to stress. By cloning stress-related transcription factor genes in maize and introducing them into maize inbred lines, the stress resistance functions of these transcription factors have been preliminarily confirmed (Figure 2). In the study of stress resistance in maize and other plants, bioinformatics methods, yeast hybridization, functional mutation analysis of transcription factors, genomics, and transcriptomics have been used to identify 56 classes of stress-related transcription factors in maize, including *AP2*, *C2H2*, *MYB*, *NAC*, *WRKY*, *ERF*, *GRAS*, *HSF*, *ARF*, *HD-ZIP*, *NF-YB*, *bZIP*, *GATA*, *NF-YA*, and *bHLH* etc. (Table 1). The *bZIP* family plays a role through the ABRE (abscisic acid-responsive element) via the ABA pathway. *WRKY* transcription factors, with over 100 members in maize, are involved in processes such as morphogenesis, metabolic regulation, seed germination, flower development, and senescence, as well as abiotic stresses, including drought, high salinity, cold damage, and oxygen deficiency due to waterlogging. In these adverse environments, WRKY proteins participate in the plant’s abiotic stress response through complex signal transduction networks. Transcription factors such as *ZmWRKY64* in maize respond to Cd stress, and these transcription factors may also play a role in the mechanism of resistance to waterlogging by regulating the expression of downstream genes to enhance plant tolerance. High salt and drought conditions can induce the expression of the transcription factor *ZmWRKY33* in maize. The overexpressed transcription factor *ZmWRKY33* activates more stress response genes, including the *RD29A* gene, thereby enhancing the plant’s tolerance to salt stress [118]. When the maize *ZmWRKY64* transcription factor was overexpressed in *Arabidopsis*, it was found that the overexpression lines affected the absorption, transport, and auxin-related genes associated with Cd stress. *ZmbHLH124* is a *bHLH* (basic helix–loop–helix) family transcription factor in maize that plays an important role in the plant’s response to drought stress. The expression of the *bHLH* family maize *ZmbHLH124* is significantly upregulated under drought stress in drought-tolerant varieties, while in sensitive varieties, there is a premature stop codon, resulting in lower expression levels. *ZmbHLH124T* can also directly activate the expression of *ZmDREB2A*, which in turn can regulate the expression of multiple genes related to stress response, thereby enhancing the plant’s tolerance to drought and heat stress [119]. *AP2/ERF* transcription factors regulate the expression of downstream genes by binding to specific cis-acting elements. Approximately 300 AP2/ERF transcription factors have been identified in maize, including 5 subfamilies: AP2 (APETALA2), ERF (ethylene response factor), DREB (dehydration response element-binding protein), RAV (related to ABI3/VP1), and Soloist. The *DREB* subfamily of transcription factors can bind to the promoters of genes containing the dehydration-responsive element (DRE), while the *ERF* subfamily of transcription factors can bind to the promoters of genes containing the GCC box. The activation of these transcription factors can enhance a plant’s tolerance to abiotic stresses such as drought, salinity stress, and extreme temperatures. The ZmEREB211 transcription factor of the maize AP2/ERF family can regulate the plant’s drought response by directly activating or suppressing the expression of downstream genes. MYB family transcription factors are also widely used in the stress resistance of maize. The MYB family of transcription factors is also widely involved in the stress resistance of maize. Haifang Jiang and colleagues used high-resolution mass spectrometry and metabolomics to reveal the relationship between natural variation in the promoter region of the cold signaling key transcription factor *ZmICE1* (Inducer of CBF Expression 1) from MYB family and maize cold tolerance. They elucidated that *ZmICE1* not only directly regulates the expression of the *DREB1* gene but also modulates the molecular mechanisms of maize’s response to low temperatures by regulating amino acid metabolism and reactive oxygen species levels [85]. *MYB* class transcription factors such as RS2 in maize [120] can be expressed in jasmonic acid responses and disease resistance, thereby regulating the expression of downstream functional genes and enhancing the plant’s disease resistance.

Of course, some transcription factors play negative roles. For example, the maize *AP2/ERF* transcription factor *ZmEREB92* has been identified as a negative regulator of maize seed germination. Transcription factors play a crucial role in plant signal transduction and stress response. Ethylene signal gene *ZmEIL7* and α-amylase gene *ZmAMYa2* have been identified as direct targets repressed by *ZmEREB92* [121]. Transcription factors, through complex regulatory networks, enable plants to adapt to changing environmental conditions. Studying these transcription factors can provide an important molecular basis for developing crop varieties with stronger stress resistance. Research on the role of transcription factors in maize stress resistance is currently very extensive and incorporates a variety of advanced technologies. However, more in-depth studies are needed to understand the mechanisms of action of transcription factors and their application in crop improvement. For example, how transcription factors interact with each other and with signaling pathways to form complex regulatory networks; the upstream regulatory factors of transcription factors; and whether the stability of transcription factor stress resistance under laboratory conditions can be ensured under field conditions.

## 3. Maize Stress-Resistance-Related Genetic Engineering

Through molecular breeding techniques, researchers have successfully cultivated some new varieties of maize with enhanced stress resistance, providing strong support for sustainable agricultural development. Genetic engineering technology involves direct manipulation of genes at the molecular level, breaking down barriers to gene exchange between different species and even completely different organisms, overcoming the obstacles of distant hybridization incompatibility, and introducing excellent genes from different sources into the recipient genome for a purpose, enriching the gene pool for resistance. The application of plant transgenic technology to improve crop varieties has shown tremendous potential, for example, introducing exogenous stress-related genes into maize to enhance its stress resistance and subsequently breed stress-resistant maize varieties. Selecting and establishing a good regeneration system is one of the key factors for the success of genetic transformation. Although various maize regeneration systems and genetic transformation systems have been established to adapt to different transformation methods and purposes, transformation methods such as gene guns and *Agrobacterium*-mediated transformation have been deeply studied, and a series of transgenic maize varieties have been put into agricultural production, the genetic transformation of maize is still very difficult, and there is no complete widely used transformation system, including in China. Therefore, strengthening research on maize regeneration systems and genetic transformation systems is of great significance [122].

### 3.1. Transgenic Breeding and Transgenic Maize Varieties

**Figure 3:**
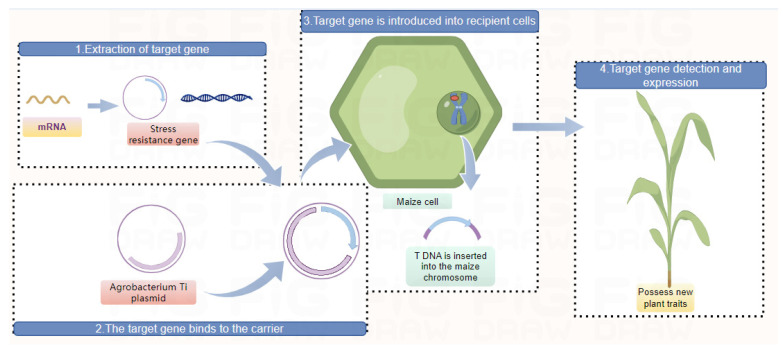
Construction process of transgenic maize.

As shown in Figure 3, genes encoding stress-resistant traits are introduced into the maize genome through transgenic technology, enabling the maize to acquire new stress-resistant characteristics. Currently, transgenic maize can be roughly divided into several categories. Insect-resistant maize: maize that can produce a protein toxic to specific pests, thereby reducing pest damage, such as by introducing genes like *cry1A.105*, *cry2Ab*, *vip3Aa20*, *mcry1Ab*, or *mcry2Ab* to form varieties resistant to Lepidopteran pests [123]. The main applications in genetically modified insect resistance in maize are Bt proteins and their recombinant proteins, including over 177 *Vip* family genes, 40 *Cyt* family genes, and 789 *Cry* family genes, among which *Cry* family genes are the most widely used in commercial genetically modified maize, followed by *Vip* family genes [124,125,126]. Drought-resistant maize: by introducing genes that enhance plant drought resistance, maize can grow better under drought conditions. For example, the MON87460 maize obtained by introducing the *cspB* gene was approved for planting in 2011 [127]. Herbicide-tolerant maize: genes that can tolerate herbicides are introduced, allowing farmers to safely use glyphosate herbicides without damaging the maize crop; examples include *bar*, *pat*, *mepsps*, *cp4-epsps*, *zm-hra*, *gat4621*, *aad-1*, *cp4-epsps*, *epspsgrg23ace5*, *2mepsps*, and *dmo* [128]. Quality-improved maize: maize’s nutritional components are improved through transgenic technology, such as increasing the content of lysine and improving the composition of fats. Environmentally adaptive maize: transgenic maize may be designed to better adapt to different climates and soil conditions. Yield-increasing maize: by improving the plant’s growth cycle, root system development, etc., transgenic maize may have higher yields. The introduction of the *athb17* gene can increase maize production; the yield-increasing maize MON87403 was approved for planting in 2015. These traits may enhance the characteristics needed for maize in agriculture [129]. Currently, one of the most widely used transgenic maize varieties is NK603; developed by the American biotechnology company Monsanto, it has been genetically modified to be resistant to glyphosate herbicides. This is one of the most representative transgenic maize varieties in the world and is also one of the most widely used. However, some countries have expressed concerns about transgenic maize. Transgenic maize MON810, also developed by Monsanto in the United States, has been banned in France and some other countries due to potential environmental hazards and impacts. However, it is still cultivated in some other countries and regions. Other transgenic maize varieties have been developed by companies such as Da Bei Nong Group and Long Ping High-tech; they have obtained variety approval and business operation permits for their transgenic maize varieties, which may have various excellent traits such as insect resistance, disease resistance, and herbicide tolerance. As of 2024, more than 70 transgenic maize varieties have been approved for commercial cultivation (Table 2) (Data from the Ministry of Agriculture and Rural Affairs of the People’s Republic of China, http://www.moa.gov.cn/, accessed on 6 November 2024).

### 3.2. New Maize Varieties Assisted by Molecular Biology Techniques

Maize varieties produced by molecular biology techniques are diverse. In addition to transgenic methods, other molecular biological means can be used to improve their stress resistance, yield, and quality. The most popular and widely followed gene tool is the CRISPR/Cas9 genome editing system, representing a new technology in the field of biological genetic manipulation following transgenic technology [130,131,132]. It can either introduce genes or not and has been applied to multiple crops. Advanced genomic techniques and quantitative genetic analysis methods can also be applied, using a strategy that combines linkage and association analysis to finely map major QTLs, clone maize functional genes, mine excellent alleles, develop functionally clear molecular markers, and apply them to the creation of new materials with excellent nutritional quality and the development of new varieties.

For instance, a team led by Professor Li Jiansheng used high-density molecular markers to successfully clone the genes controlling the content and composition of provitamin A and vitamin E in maize kernels and designed multiple functional molecular markers, ultimately breeding several new varieties of maize high in provitamin A and vitamin E, paving a new way for maize quality breeding [133]. Through molecular marker-assisted selection, the excellent allele gene *crtRB1* controlling the synthesis of provitamin A has been successfully cloned, and related functional markers have been developed [134]. Professor Yan Jianbing’s team at Huazhong Agricultural University used the maize database (ZEAMAP) for research and successfully increased the vitamin E content in sweet maize by more than 17%, with the highest increase reaching 2.5 times. Maize obtained through transgenic technology has significantly increased α-tocopherol content and phytase activity in the kernels, providing a material basis for feed maize breeding. More than 90% of γ-tocopherol in transgenic maize kernels is converted into α-tocopherol, with the content of α-tocopherol reaching 50–70 mg·kg^−1^, which is enough to fully meet the needs of animal feed.

New varieties of high-oil maize: These were also developed by Professor Li Jiansheng’s team through techniques such as whole-genome association analysis. During the process of breeding new varieties of maize high in provitamin A and vitamin E, Professor Li Jiansheng’s team also discovered genes affecting the oil content and the composition and ratio of fatty acids in maize through whole-genome association analysis and proposed the theory that “the accumulation of excellent alleles is an important genetic basis for the increase in maize oil content [135]”. They mined excellent alleles affecting oil content and composition and developed corresponding functional molecular markers. By using these markers, excellent high-oil alleles were successfully introduced into widely cultivated hybrid maize varieties in China, significantly increasing the oil content of maize. In this process, the team also used gene editing technology to create new materials with the functional knockout of the *KRN2* and *OsKRN2* genes, which can increase the yield of maize and rice. Years of field plot trials have shown that by knocking out the *KRN2* and *OsKRN2* genes through gene editing, maize yield can be increased by about 10% and rice yield by about 8% without significant negative impacts on other agronomic traits [136].

These research achievements not only enrich the theory of crop domestication genetics but also have significant implications for further improving the efficiency of crop breeding, making important contributions to the quality improvement of agricultural products, and the development of agricultural science and technology. Genes applied through gene editing in maize include regulating male sterility: *ZmHLH51*, *ZmbHLH122*, *ZmTGA9-1*, *ZmTGA9-2*, *ZmTGA9-3*, *ZmTGA10*, *ZmMYB84*, *ZmMYB33-1*, *ZmMYB33-2*, *ZmPHD11*, *ZmLBD10*, *ZmLBD27*, *ZmDFRI/2*, *ZmCO1*, *ZmTKPRI-I*, and *ZmTKPRI-2* [137]; pollen development: *ZmC012a*, *ZmCO12b*, and *ZmPKSB*; haploid induction rate: *ZmDMP* and *ZmPLD3*; maize kernel aroma: *ZmBADH2a* and *ZmBADH26*; maize waxy texture: *ZmWx1*; maize kernel sweetness: *ZmSHRUNKEN2*; kernel size: *ZmCEP1*; flowering: *ZmPHYC1*, *ZmPHYC2*, and *ZmCCT9*; tassel branch number: *ZmPAT7*; plant height: *ZmCEPI* and *ZmGA200x3*; ear axis length: *ZmCLE* and *ZmYIGEI*; leaf angle: *ZmLG1*; lateral root number: *ZmIRT1*; drought resistance: *ZmARGOS8*, *ZmGA20ox3*, and *ZmBETSL1*; salinity–alkalinity tolerance: *ZmEREB57*, and *ZmAT1*; water use efficiency: *ZmAbh4*; other stress tolerances: *ZmAbh4*, *ZmLOX3*, *ZmG6PDH1*, *ZmChSK1*, and *ALSase*. These genes play crucial roles in various aspects of maize development and stress responses, and their manipulation through gene editing technologies has the potential to significantly enhance the traits of interest in maize breeding programs.

In the future, transgenic breeding should focus on utilizing molecular-assisted markers, whole-genome technologies, gene editing technologies, and combining artificial intelligence + big data to strengthen the exploration and mining of new genes related to yield, quality, pest and disease resistance, drought tolerance, salinity–alkalinity tolerance, and health functions. This approach aims to obtain more new genes with independent intellectual property rights and industrial application value for important traits, providing excellent genetic resources for novel variety breeding.

## 4. Conclusions and Prospects

This paper reviews the physiological and biochemical responses, as well as the molecular mechanisms, of maize when facing adverse conditions such as high temperatures and drought, low temperatures, heavy metals, salinization, and diseases. Research results indicate that maize responds and adapts to different stress conditions through a series of complex signal transduction pathways and the regulation of transcription factors, such as NPR1, the WRKY family, and the HD-Zip family members. In addition, the application of GWAS (Genome-Wide Association Studies) and gene editing technologies like the CRISPR/Cas system provides new strategies and tools for the genetic improvement of maize’s stress-resistance traits. By conducting an in-depth analysis of the molecular mechanisms of maize’s stress response, we can better understand these processes and provide new perspectives for future research. Future research can further explore the functions of key genes in stress signal transduction pathways and how to cultivate new varieties of maize with greater stress resistance through gene editing technologies. This will provide important scientific evidence for improving crop tolerance using molecular biology methods. Future research will continue to advance the development of this field and make greater contributions to agricultural production and food security. Despite extensive research on maize stress resistance at the molecular level, integrating and analyzing multi-omics data effectively, as well as organically combining these data with phenotypic changes, remains a challenge. Additionally, crops modulate the expression of stress-resistance genes through various signaling pathways when facing complex environmental stresses, and our understanding of these complex regulatory networks is still limited. Moreover, although gene editing technology has provided significant support for crop genetic improvement, improving editing efficiency and precision is still a pressing issue to be addressed.

Prospects and suggestions for future molecular research on maize stress resistance include.

A focus on multi-omics association analysis has become a common technical means for studying crop stress responses. By integrating genomics, transcriptomics, proteomics, metabolomics, etc., a more comprehensive understanding of the molecular response mechanisms of crops under stress can be achieved, and bioinformatics tools can be used for data integration and analysis.

Intelligent plant type research: How to achieve coordinated morphological improvement and physiological adaptation through the study of intelligent strains of maize, promoting the increase in maize yield through dense planting.

Pay attention to the impact of environmental changes on maize, particularly its response mechanisms to new environmental stresses (such as extreme weather events, including high or low temperatures and drought). Continue to delve into the response mechanisms of maize to such abiotic stresses, especially the molecular mechanisms in relation to transcription factors, protein modifications, and reactive oxygen species balance.

Finally, it strengthens the combination of basic research and applied research, quickly transforms basic research results into practical breeding applications, and improves the production efficiency and stress resistance of maize. Gain a deep understanding of the molecular mechanisms of maize–pathogen interactions, discover resistance-related genes, and provide a theoretical basis for the cultivation of disease-resistant varieties.

## Figures and Tables

**Table 1:** Maize transcription factors by family.

Family	Family	Family	Family
*AP2*	*Dof*	*LBD*	*RAV*
*ARF*	*E2F*	*LFY*	*S1Fa-like*
*ARR-B*	*EIL*	*LSD*	*SBP*
*B3*	*ERF*	*MIKC*	*SRS*
*BBR*	*FAR1*	*M-type*	*STAT*
*BES1*	*G2-like*	*MYB*	*TALE*
*bHLH*	*GATA*	*MYB-related*	*TCP*
*bzip*	*GeBP*	*NAC*	*Trihelix*
*C2H2*	*GRAS*	*NF-X1*	*VOZ*
*C3H*	*GRF*	*NF-YA*	*Whirly*
*CAMTA*	*HB-others*	*NF-YB*	*WOX*
*CO-like*	*HD-ZIP*	*NF-YC*	*WRKY*
*CPP*	*HRT-like*	*Nin-like*	*YABBA*
*DBB*	*HSF*	*PHD*	*ZF-HD*

**Table 2:** Transgenic maize transformants approved for commercial planting.

Trait	Transformed Name	Research Institute or Company	Target Gene	Year of Approval
Resistant to Lepidopteran pests	MON801	Monsanto (Creve Coeur, MO, USA)	*cry1Ab*	1995
	MON809	Monsanto (Creve Coeur, MO, USA)	*cry1Ab*	1996
	MON810	Monsanto (Creve Coeur, MO, USA)	*cry1Ab*	1996
	MON802	Monsanto (Creve Coeur, MO, USA)	*cry1Ab*	1997
	MON89034	Monsanto (Creve Coeur, MO, USA)	*cry1A.105*, *cry2Ab*	2008
	MIR162	Syngenta Group (Basel, Switzerland)	*vip3Aa20*	2009
	ND207	China Agricultural University, China National Tree Seed Group Corporation Limited (Beijing, China)	*mcry1Ab*, *mcry2Ab*	2021
	RF8	RFGENE, Zhejiang University (Hangzhou, China)	*cry1Ab*, *cry2Ab*	2021
	MON 95379	-	*Cry1Ab*, *Cry1b.868*, *Cry1be*, *Cry1ca*, *Cry1da_7*	2023
Resistant to Coleopteran pests	MON863	Monsanto (Creve Coeur, MO, USA)	*cry3Bb1*	2002
	MIR604	Syngenta Group (Basel, Switzerland)	*mcry3A*	2007
	5307	Syngenta Group (Basel, Switzerland)	*ecry3.1Ab*	2013
	MON 95275	-	*Mpp75Aa1.1*, *Vpb4Da21*, *DvSnf7.1*	2023
Herbicide-tolerant	DLL25 (B16)	Monsanto (Creve Coeur, MO, USA)	*bar*	1995
	T14	Bayer (Berlin, Germany)	*pat*	1995
	T25	Bayer (Berlin, Germany)	*pat*	1995
	GA21	Monsanto (Creve Coeur, MO, USA)	*mepsps*	1997
	NK603	Monsanto (Creve Coeur, MO, USA)	*cp4-epsps*	2000
	98140	DowDuPont (Wilmington, DE, USA)	*zm-hra*, *gat4621*	2009
	DAS40278-9	Dow Agrosciences China Ltd. (Beijing, China)	*aad-1*	2012
	MON87427	Monsanto (Creve Coeur, MO, USA)	*cp4-epsps*	2012
	VCO01981-5	Genective (Weldon, IL, USA)	*epspsgrg23ace5*	2013
	MZHG0JG	Syngenta Group (Basel, Switzerland)	*2mepsps*, *pat*	2015
	MON87419	Monsanto (Creve Coeur, MO, USA)	*dmo*, *pat*	2016
	MON87429	Monsanto (Creve Coeur, MO, USA)	*cp4-epsps*, *dmo*, *pat*, *ft_t*	2020
	DBN9858	Da Bei Nong Group (Beijing, China)	*EPSPS*, *pat*	2022
	nCX-1	RFGENE (Hangzhou, China)	*CdP450*, *cp4epsps*	2022
	GA21	China National Seed Group Co., Ltd. (Beijing, China)	*mepsps*	2022
	DP-2Ø2216-6	Corteva (Indianapolis, IN, USA)	*pat*	2024
	DP202216 × NK603 × DAS40278	Bayer (Berlin, Germany)	*-*	2024
	DP-2Ø2216-6 × MON-ØØ6Ø3-6 × DAS-4Ø278-9	Corteva (Indianapolis, IN, USA)	*pat*, *epsps*, *aad-1*	2024
Drought-resistant	MON87460	Monsanto (Creve Coeur, MO, USA)	*cspB*	2011
Male sterility	MS3	Bayer (Berlin, Germany)	*barnase*	1996
	MS6	Bayer(Berlin, Germany)	*barnase*	1999
	DP32138-1	DowDuPont (Beijing, China)	*ms45*, *zm-aa1*	2011
Quality improvement	LY038	Monsanto (Creve Coeur, MO, USA)	*cordapA*	2006
	3272	Syngenta Group (Basel, Switzerland)	*amy797E*	2008
	BVLA430101	Origin Agritech (Beijing, China)	*phyA2*	2009
	PY203	Agrivida (Woburn, MA, USA)	*phy02*, *pmi*	2021
	MON94804	Bayer (Berlin, Germany)	*GA20ox_SUP*	2024
	DP910521	-	*-*	2024
Yield increase	MON87403	Monsanto (Creve Coeur, MO, USA)	*athb17*	2015
Resistant to Lepidopteran pests and herbicides	Bt176	Syngenta Group (Basel, Switzerland)	*cry1Ab*, *bar*	1995
	Bt11	Syngenta Group (Basel, Switzerland)	*cry1Ab*, *pat*	1996
	DBT418	Monsanto (Creve Coeur, MO, USA)	*cry1Ac*, *bar*, *pinI*	1997
	CBH351	Bayer (Berlin, Germany)	*cry9C*, *bar*	1998
	TC1507	Dow Agrosciences China Ltd., DowDuPont (Beijing, China)	*pat*, *cry1Fa2*	2001
	TC6275	Dow Agrosciences China Ltd. (Beijing, China)	*bar*, *mocry1F*	2004
	EH913	Helix (San Diego, CA, USA)	*cry1Da_7*	2024
	DP910521	Corteva (Indianapolis, IN, USA)	*cry1B.34*, *PAT*	2024
	DP-91Ø521-2	Corteva (Indianapolis, IN, USA)	*Cry1B.34*, *PMI*, *PAT*	2024
Resistant to Coleopteran pests and herbicides	59122	Dow Agrosciences China Ltd., DowDuPont (Beijing, China)	*cry34Ab1*, *cry35Ab1*, *pat*	2005
	MON88017	Monsanto (Creve Coeur, MO, USA)	*cry3Bb1*, *cp4-epsps*	2005
	MON87411	Monsanto (Creve Coeur, MO, USA)	*cry3Bb1*, *cp4-epsps*, *dvsnf7*	2015
	MZIR098	Syngenta Group (Basel, Switzerland)	*ecry3.1Ab*, *mcry3A*, *pat*	2016
	YN-E3272-5 × SYN-BTØ11-1 × SYN-IR162-4 × MON-ØØØ21-9	-	*cry1Ab*, *EPSPS*, *pat*	2023
	SYN-E3272-5	-	*amy797E*	2023
	DP23211	Corteva (Indianapolis, IN, USA)	*pat*, *pmi*	2024
Resistant to both Lepidopteran and Coleopteran pests, and herbicides	4114	DowDuPont (Wilmington, DE, USA)	*cry1F*, *cry34Ab1*, *cry35Ab1*, *pat*	2013
	RF125	RFGENE, Zhejiang University (Hangzhou, China)	*cry1Ab*/*cry2Aj*, *g10evo-epsps*	2019
	DBN9936	Da Bei Nong Group (Beijing, China)	*cry1Ab*, *epsps*	2019
	DBN9501	Da Bei Nong Group (Beijing, China)	*vip3Aa19*, *pat*	2020
	DBN3601T(DBN9936 × DBN9501)	Da Bei Nong Group (Beijing, China)	*cry1Ab*, *epsps*, *vip3Aa19*, *pat*	2021
	1507 × MIR162 × MON810 × NK603	-	*-*	2021
	DP-ØØ4114-3	-	*cry1F*, *cry34Ab*, *cry35Ab1*, *pat*	2022
	Bt11 × GA21	China National Seed Group Co. Ltd. (Beijing, China)	*cry1Ab*, *pat*, *mepsps*	2022
	Bt11 × MIR162 × GA21	China National Seed Group Co. Ltd. (Beijing, China)	*cry1Ab*, *pat*, *vip3Aa20*, *mepsps*	2022
	3272 × Bt11 × MIR162 × MIR604 × TC1507 × 5307 × GA2	-	*-*	2023
Male sterility and herbicide tolerance	676	DowDuPont (Wilmington, DE, USA)	*pat*, *dam*	1998
	678	DowDuPont (Wilmington, DE, USA)	*pat*, *dam*	1998
	680	DowDuPont (Wilmington, DE, USA)	*pat*, *dam*	1998
Herbicide tolerance and yield increase	DP202216	Dow Agrosciences China Ltd. (Nantung, China)	*zmm28*, *mo-pat*	2020
Resistant to Maize rootworm	MON95275	Bayer (Berlin, Germany)	*Mpp75Aa1.1*, *Vpb4Da2*, *Dvsnf7.1*	2024
Resistant to Maize rootworm and herbicides	DP915635	Corteva (Indianapolis, IN, USA)	*pat*, *IPD079Ea*, *pmi*	2024
Insect-resistant and drought-resistant	SAMMAZ 72T	-	-	2024
	SAMMAZ 73T	-	-	2024
	SAMMAZ 74T	-	-	2024
	SAMMAZ 75T	-	-	2024
Insect-resistant, drought-resistant, and herbicide-tolerant	MON87427 × MON87460 × MON89034 × 1507 × MON87411 × 59122	-	-	2021

## Data Availability

Not applicable.
